# Presumed Alcohol-Induced Ventricular Tachycardia Storm: A Case Report

**DOI:** 10.7759/cureus.8097

**Published:** 2020-05-13

**Authors:** Gian Lima, Eduardo Cardoso, Garret Fiscus

**Affiliations:** 1 Internal Medicine, University of Connecticut Health Center, Hartford, USA; 2 Department of Internal Medicine, University of Connecticut, Hartford, USA

**Keywords:** alcohol misuse, cardiac arrest, out of hospital cardiac arrest, sudden death, ventricular tachycardia

## Abstract

Alcohol abuse is a widely recognized cause of supra-ventricular fibrillation, but in some patients, it is also associated with ventricular arrhythmias and even sudden death. We describe a case of a 36-year-old patient who, with no risk factors for coronary disease and with a structurally normal heart, experienced two episodes of cardiac arrest five years apart, with both events occurring after significant alcohol consumption. It is important to recognize that the prognosis of alcohol-induced arrhythmias is usually good in patients who remain compliant with alcohol cessation and to avoid the misdiagnosis of “idiopathic” ventricular tachycardia/ventricular fibrillation (VT/VF).

## Introduction

Alcohol abuse is a well-known cause of atrial fibrillation, but in some patients, it is also associated with ventricular arrhythmias and even sudden death. Even though 86% of the people aged 18 or older in the US report they have consumed alcohol at some point in their life and sudden cardiac death is the leading cause of mortality worldwide (accounting for an estimated 15-20% of all deaths), alcohol-induced cardiac arrest is an underrecognized condition [1,2]. We describe a case of a young patient who, with a structurally normal heart, experienced two episodes of cardiac arrest after significant alcohol consumption.

## Case presentation

The patient was a 36-year-old male with a history of cardiac arrest five years earlier, status post-implantable cardioverter-defibrillator (ICD) placement with no other comorbidities; the patient was not on chronic medications. He presented to the emergency department (ED) in the morning complaining of palpitations and three ICD shocks overnight. He admitted that he had consumed six drinks containing vodka the night before, but stated that he only drank sporadically and rarely had more than one drink. The patient reported palpitations prior to the ICD shock and mild chest pain after the shock delivery but denied shortness of breath, dizziness, and loss of consciousness.

In the ED, his initial vital signs were as follows: blood pressure of 120/75 mmHg, heart rate of 85 bpm, afebrile, and oxygen saturation of 98% on room air. He was initially placed on telemetry, which showed atrial fibrillation with heart rate in the 90s; however, subsequent 12-lead electrocardiogram (EKG) demonstrated that he had spontaneously converted into sinus rhythm, with no ST deviations and a QTc of 461 msec. Additionally, there were no significant laboratory abnormalities (complete blood count within normal limits, Mg 2.2 mEq/L, K 3.6 mEq/L, Ca 9.6 mg/dL, Phos 3.4 mg/dL, Cr 1.0 mg/dL); troponin was negative (ref <0.3 ng/mL), and urine toxicology was negative for cocaine, amphetamines, barbiturates, cannabinoid, and opiates. The ethanol level was not checked.

**Figure 1:**
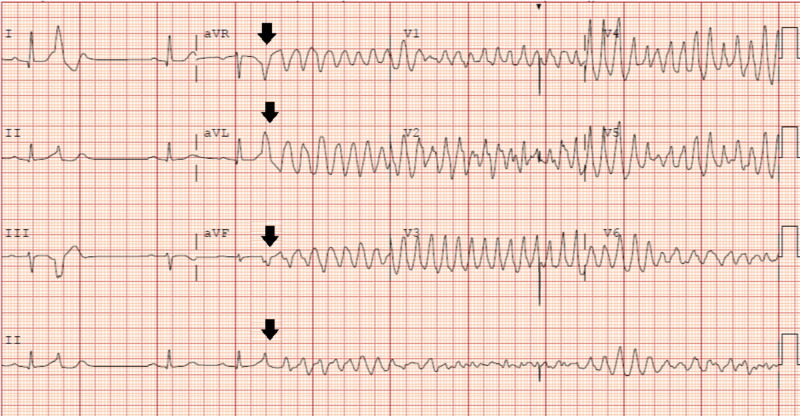
EKG on admission EKG demonstrating a premature ventricular beat preceding the polymorphic ventricular arrhythmia. Prior to PVC, the QTc interval was within normal limits. The black arrows indicate the PVC occurring during the T wave, characterizing the R-on-T phenomenon EKG: electrocardiogram; PVC: premature ventricular complex

While in the ED, the patient was found to have multiple episodes of non-sustained ventricular tachycardia (VT) on telemetry, which resulted in another ICD shock. He was admitted to the cardiac care unit (CCU) and given lidocaine bolus 100 mg, followed by continuous infusion at 2 mg/min. The patient continued to have multiple episodes of VT and ICD shocks, and amiodarone infusion was started at 1 mg/min after an initial bolus of 150 mg. Because of the increasing distress and agitation, the patient was sedated and intubated. Despite dual anti-arrhythmic therapy, he continued to have VT storm and received approximately 25 ICD shocks in two hours, followed by ventricular fibrillation (VF), requiring external defibrillation and cardio-pulmonary resuscitation for eight minutes. A review of the patient’s EKG during VT storm indicated premature ventricular complex (PVC) triggering polymorphic VT with the R-on-T phenomenon (Figure 1).

Given his unstable condition, a short-term external circulatory assist device was placed, and he was started on extracorporeal membrane oxygenation (ECMO) support. He continued on amiodarone drip and temperature control through the ECMO circuit thermoregulator. No recurrent episode of VT/VF or ICD shock was noted after the ECMO placement. Initial transthoracic echocardiogram (TTE) showed severe global hypokinesis (ejection fraction of 20%), with no regional wall motion abnormality. The patient initially required norepinephrine and dobutamine for pressor and inotropic support but was able to wean off vasopressors, sedation, and he tolerated ECMO flow reduction overnight. A Repeat TTE performed one day later showed normal left ventricular function, making ECMO withdrawal possible on the third day of admission. The patient was subsequently extubated and found to have no residual neurological deficits. Amiodarone was discontinued, and he was started on oral flecainide 100 mg twice daily and diltiazem 120 mg daily for PVC suppression. He remained in sinus rhythm, with no ventricular ectopy on telemetry.

A cardiac MRI was performed and showed no late post-gadolinium myocardial enhancement suggestive of myocardial fibrosis and no evidence of structural abnormalities; however, the test was suboptimal because of artifacts. A cardiac positron emission tomography (PET) scan demonstrated a structurally normal heart, with no evidence of infiltrative or inflammatory disease. As there was no ventricular ectopy on telemetry for several days, it was determined that an electrophysiology study would likely not be beneficial. The patient was discharged after 10 days of hospital course.

**Figure 2:**
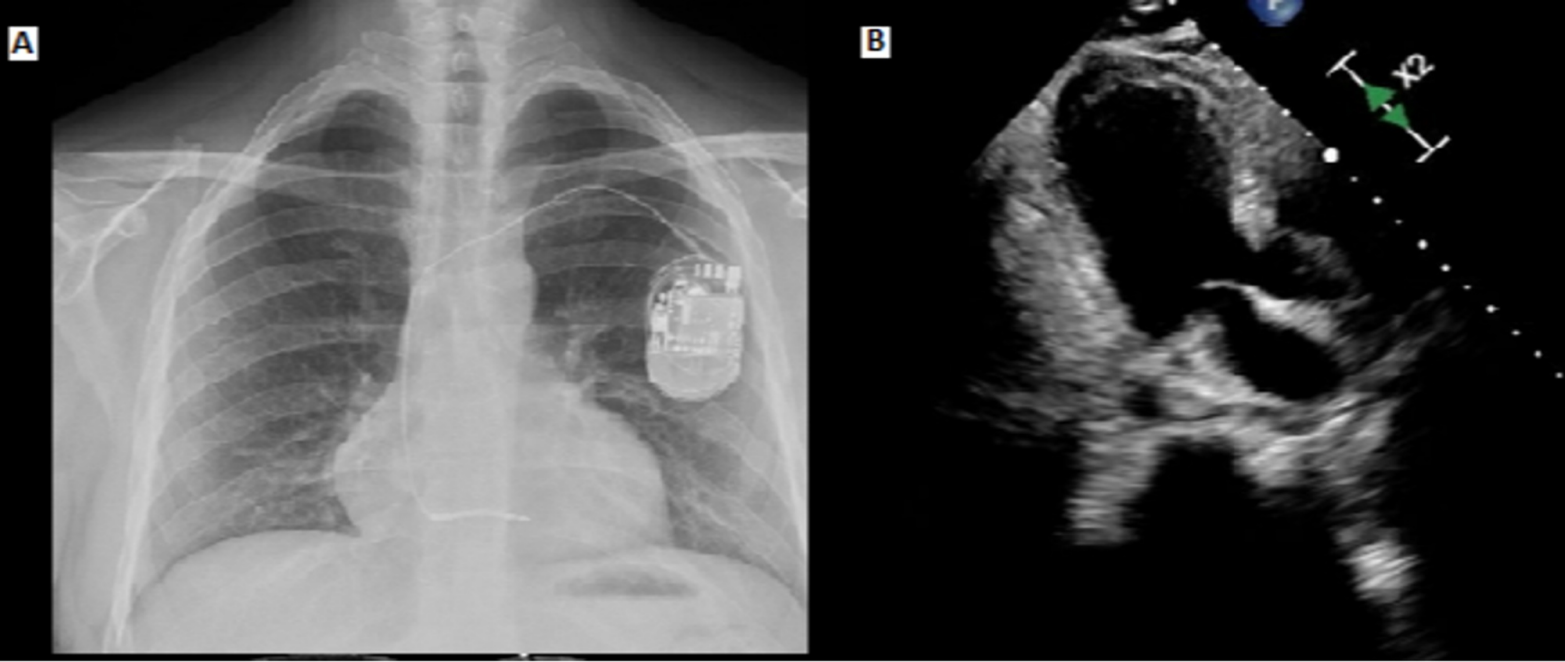
Cardiac imaging on admission A: chest radiograph demonstrated no acute cardiopulmonary disease. B: TTE - left ventricular function was severely globally reduced (20%); a repeat TTE performed one day later showed that left ventricular function had recovered TTE: transthoracic echocardiogram

Of note, the patient had experienced an earlier similar episode of VT/VF cardiac arrest at 31 years of age. At that time, the patient had drunk at least four drinks with vodka the night before the event, which he described as an unusual amount of alcohol for him. After his wife had witnessed him collapsing in the morning, EMS had been contacted, and he had been successfully resuscitated after seven defibrillations and a total on-scene time of approximately 38 minutes. Subsequent in-hospital workup had shown normal coronary arteries on angiography and a structurally normal heart with an ejection fraction of 60% on TTE. One of the patient's EKGs demonstrated a prolonged QTc (540 msec), and he had an ICD implanted for secondary prevention prior to discharge. As an outpatient, he had a genetic test for long QTc syndrome, reportedly inconclusive. No history of early coronary disease, cardiomyopathy, or sudden death in his family had been reported. He has two children who also underwent a genetic test for long QT, both negative. Upon follow-up, the patient’s EKG had shown a QTc of 428 msec. The patient had never experienced an episode of ICD shock until his presentation to ED five years later.

## Discussion

In this report, we discussed a previously healthy young male with no risk factors for coronary artery disease who most likely had VT/VF triggered by alcohol consumption. The patient experienced two episodes of VT, five years apart, with highly similar presentations: he started experiencing palpitations in the morning after excessive alcohol consumption and was initially found to have atrial fibrillation in the ED (which is the most common arrhythmia related to increased alcohol intake) and subsequently had VT. After the workup ruled out electrolyte abnormalities, coronary artery disease, and structural heart disease, and considering the history of significant alcohol use prior to both events, we presumed that the patient had alcohol-induced cardiac arrest.

The adverse relationship between alcohol and heart diseases has been well described in the literature. In 1978, Ettinger et al. used the term “holiday heart” to describe acute arrhythmias (most commonly atrial fibrillation but also atrial flutter, PVC, and VT) following heavy alcohol consumption in patients without preexisting cardiac disease with normalization of the rhythm with avoidance of alcohol [3]. Large epidemiologic studies have shown association between heavy alcohol consumption (three to five drinks/day) and VT/sudden cardiac death, though a lower rate of VT/VF was demonstrated in patients with lower alcohol intake (two to six drinks/week), likely due to the protective effects of low-moderate alcohol ingestion on the risk of coronary artery disease [4].

Despite data from the World Health Organization showing that alcohol abuse causes about 2.5 million deaths annually, alcohol-induced cardiac arrest is still underrecognized as many of these cases are incorrectly attributed to minor coronary lesions or simply classified as “idiopathic.” In a UK survey of sudden deaths involving 1,292 postmortem analyses, 1.2% had no clear cause of death and a history of alcohol abuse, suggesting that a proportion of sudden deaths can be due to alcohol-related arrhythmias, which was often unrecognized [5].

The mechanisms that contribute to the development of arrhythmias likely involve intramyocardial and adrenal release of catecholamines, increased acetaldehyde levels, deranged plasma electrolytes (most commonly magnesium and potassium), and abnormal autonomic nervous system discharges [6-9]. Moreover, it has been proposed that high serum ethanol levels can cause the prolongation of PR, QRS, and QT intervals, possibly due to interference with sodium, potassium, and calcium ion channels in the heart, and these changes can sensitize the myocardium to atrial and ventricular arrhythmias [10,11].

## Conclusions

It is important to recognize that the prognosis for alcohol-induced arrhythmias is usually good in patients who remain compliant with alcohol cessation. This must be considered as a critical part of the therapy and often accorded more importance than the prescription of anti-arrhythmic agents. Therefore, it is fundamental to identify these patients and avoid the misdiagnosis of “idiopathic” VT/VF since simple lifestyle changes can result in significantly better outcomes in this group of patients.
